# Motivation regulation strategies and their role in resolving motivational conflicts: evidence from two scenario studies from students’ daily life

**DOI:** 10.3389/fpsyg.2026.1850323

**Published:** 2026-06-18

**Authors:** Lisa Otte, Gabriele Steuer, Nina Brassler, Lea Adam, Stefan Fries

**Affiliations:** Department of Psychology, University of Bielefeld, Bielefeld, Germany

**Keywords:** higher education, motivational conflict, motivational interference, motivational regulation, self-regulated learning

## Abstract

Previous studies have demonstrated that motivational regulation strategies (MRS) are effective in addressing and overcoming motivational problems. In the present empirical studies, the use of MRS in a motivational conflict and their relationship with the experience of motivational interference are examined. In two online studies (Study 1: *N* = 214 and Study 2: *N* = 242), students were presented with a motivational conflict scenario. Subsequently their use of specific MRS, including the use of motivation regulation strategies and quality of their use, as well as their experience of motivational interference was assessed via questionnaires. Results showed that both the use of motivation regulation strategies and application quality of MRS were associated with lower motivational interference. Certain strategies were associated with less interference, though not all MRS had a significant impact. These findings suggest that students can self-regulate their experience of motivational interference and highlight the potential for developing self-regulation skills in educational contexts, including new strategies alongside existing ones.

## 1 Introduction

Both research on motivational conflicts and motivational regulation strategies has become increasingly important in recent years, as it offers crucial insights into the behavior and decision-making of individuals. Motivational conflicts, which occur when the motivation toward one goal collides with the motivation for another goal, can have a significant impact on motivation to learn (Dietz et al., 2005). While numerous studies have examined the occurrence, nature, and consequences of motivational conflicts, comparatively little is known about how individuals can effectively cope with such conflicts. Research on motivational regulation strategies provides a promising starting point for addressing this question. While these strategies have primarily been studied in the context of motivational problems related to expectancy and value, it remains unclear whether they are also effective in situations characterized by motivational conflicts. A recent systematic review documents the state of research on motivation regulation (Trautner et al., 2025) and emphasizes its significance in the context of higher education. The scoping review identifies areas where further research is needed. Students often have to decide between various learning and leisure activities, leading to motivational conflicts (Grund et al., 2015b). To date, there has been little research on whether motivation regulation strategies are associated with more adaptive outcomes in the situation of a motivational conflict, too. Initial findings by a longitudinal study by Kim and Yu (2024) showed that motivational regulation strategies can be beneficial in dealing with motivational conflicts. However, further research is needed to better understand the relevance of these strategies with a special focus on conflict-related strategies on the experience of motivational conflict. The present studies were conducted to gain insights into the role of motivational regulation strategies in situations of motivational conflict.

### 1.1 Motivational conflicts and motivational interference

Motivational conflicts refer to situations in which multiple action tendencies are simultaneously valued but cannot be pursued at the same time (Hofer, 2004). In such situations, individuals are confronted with competing goals, such as studying versus engaging in a leisure activity (e.g., Fries et al., 2008; Grund et al., 2015a; Hofer and Fries, 2016). Motivational conflicts are ubiquitous in students’ everyday lives, as they often pursue different goals, with their extracurricular goals frequently influencing their learning (Dowson and McInerney, 2003; Grund et al., 2015b; Wentzel, 2003). In many situations, they do not have enough resources to pursue the different competing goals (Grund et al., 2015b; Hofer, 2004). When motivational conflicts persist after deciding for one and against another action, they can still impair the execution of the chosen action, which is called motivational interference (Fries, 2006).

Thus, *motivational interference* is defined as impairments in the experience and behavior in the current action due to motivational characteristics of unchosen alternatives (Fries, 2006). It manifests in bad mood, higher distractibility, switching, thoughts about the missed alternative, and reduced depth of processing during learning (e.g., Brassler et al., 2021; Hofer et al., 2017). Incentives of the unchosen action (e.g., meeting friends) are referred to as opportunity costs for learning (Grund and Fries, 2012), as they represent the benefits that must be given up in order to engage in the focal activity.

If the incentive of the missed leisure activity is higher, the interference becomes more pronounced in terms of affective, cognitive, and behavioral symptoms as mentioned above (Schmid et al., 2005). Conversely, unchosen but important learning activities can also dampen the enjoyment of leisure (Hofer et al., 2017).

Taken together, motivational conflicts may substantially impair students’ learning motivation due to the presence of competing alternatives. Therefore, motivational conflicts are a phenomenon which is important to consider when dealing with learning motivation. Nonetheless, previous research on motivational conflicts and motivational interference has predominantly focused on the evidence and description of the phenomenon, its effects on experience and behavior and on the explanation of differences in motivational interference related to situational characteristics of the motivational conflict, such as value and expectancy of the focal and concurring alternative (Brassler et al., 2016; Dietz et al., 2005; Fries and Schmid, 2007; Fries et al., 2008; Grund et al., 2014, 2015b). Only little research has addressed the question of how to reduce motivational interference in learning (Kim et al., 2023; Kim and Yu, 2024).

### 1.2 Motivational regulation

Regulation of motivation comprises all actions of a learner that are used to initiate, maintain and increase the motivation (Wolters, 2003) and are supposed to enhance persistence and effort in learning activities (e.g., Schwinger et al., 2009; Wolters, 2011; Zimmerman, 2011). As such motivational regulation can be referred to as a crucial component of self-regulated learning (Boekaerts, 1999). In their Motivational Regulation Model Schwinger and Stiensmeier-Pelster (2012) described the process of motivational regulation: Students have to recognize an undesired drop of motivation and to identify a motivational problem that caused it, e.g., working on a boring topic. In order to increase motivation again students may now choose one (or more) motivation regulation strategy and apply it in the next step (e.g., deliberately identifying potential applications of the topic in their own life, such as considering how the content might be relevant for their future career). For the effectiveness of strategy use not only the appropriateness of the strategy is relevant but also the application quality of strategy (Engelschalk et al., 2017). Successful strategy selection and application results in increased levels of motivation - manifested e.g., in higher effort and persistence (Schwinger and Stiensmeier-Pelster, 2012).

#### 1.2.1 Motivational regulation strategy use

Strategies are generally defined as sequences of actions that facilitate achieving specific goals (Alexander et al., 1998). For motivational regulation strategies this goal is to manipulate the motivation. The use of motivational regulation strategies can vary widely in terms of which strategies are chosen and how frequently they are applied in order to regulate study motivation (Schwinger et al., 2012). Notable strategies in the literature (e.g., Engelschalk et al., 2015; Schwinger et al., 2009; Wolters, 1998, 1999) include: *enhancing personal relevance* (connecting study material with personal goals), *enhancing of situational interest* (modifying tasks to be more engaging), *mastery self-talk* (focusing on the aim of improving one’s competence), *performance-approach self-talk* (emphasizing positive outcomes of learning), *performance-avoidance self-talk* (highlighting negative consequences of underperformance), *self-consequating* (linking achieved goals with rewards), *proximal goal setting* (breaking tasks into manageable steps), *ability-focused self-talk* (recognizing personal strengths or past successes), and *environmental control* (minimizing distractions).

Research in higher education empirically links the use of motivational regulation strategies with enhanced motivation like increased study effort and persistence (e.g., Schwinger et al., 2009; Wolters, 1998, 1999, 2003). It is also associated with more effective motivational regulation, which is connected with multiple other favorable aspects of learning processes (Eckerlein et al., 2019; Schwinger and Stiensmeier-Pelster, 2012).

But using MRS itself is not sufficient. Also, application quality of MRS plays a crucial role for successful motivational regulation. Application quality of is characterized by deploying strategies in a way that they effectively initiate, maintain or increase study motivation (Engelschalk et al., 2017). This involves metacognitive processes such as planning, executing, monitoring, and adjusting strategy use as necessary. To maximize the effectiveness of self-regulation, strategies need to be applied accurately, cohesively, and consistently (e.g., Pintrich, 2005; Winne and Hadwin, 2008; Zimmerman, 2000). For example, a student tackling complex material might initially set broad goals but, upon monitoring their progress, realize these aren’t sustaining their motivation. They might then adjust their approach by setting smaller, more attainable goals to enhance motivation and regulatory effectiveness (see Engelschalk et al., 2017). In an experimental study by Leutner et al. (2001), students were taught to enhance personal significance within a digital learning environment. One group received basic strategy training, while another group also learned how to monitor and regulate their strategy use. Both groups outperformed a control group, but the latter exhibited greater motivation and learning improvements, highlighting the importance of high-quality strategy use. Furthermore, a longitudinal study by Eckerlein et al. (2019) demonstrated that high-quality motivational regulation helped maintain study effort over a 14-day exam preparation period, with both the use of motivational regulation strategies and quality positively affecting exam performance.

Thus, both the use and the quality of motivational regulation strategies contribute to enhanced motivation.

### 1.3 Specific motivational problems

Recent research on motivational regulation has begun to focus on the conditions under which particular strategies are most effective, by distinguishing between different types of motivational problems (Eckerlein et al., 2022). A common distinction is made between expectancy-related problems, such as low success expectations, and value-related problems, such as a perceived lack of interest or personal relevance. Empirical findings suggest that students select different strategies depending on the nature of the problem, and that the fit between strategy and situation is critical for regulatory success (Steuer et al., 2019). The motivational problems examined in this line of research have almost exclusively addressed problems that arise from characteristics of the focal learning activity. Given that motivational conflicts are a common feature of students’ everyday lives and have been shown to be associated with impairments in functioning, it seems reasonable to consider them as a specific type of motivational problem. In motivational conflicts, the challenge does not arise from the focal task itself, but from the simultaneous presence of an attractive alternative.

However, this perspective has not yet been systematically addressed in previous research. Motivational conflicts place particular demands on learners’ self-regulation, as they require managing competing action tendencies during ongoing learning activities.

### 1.4 Motivational regulation strategies as tools for overcoming motivational interference

Motivational conflicts and motivational interference are highly relevant in higher education contexts, as university students frequently face competing academic and non-academic demands (e.g., Kim and Seo, 2018). Building on motivational regulation research, the present studies conceptualize motivational conflicts as a specific type of motivational problem that may trigger motivational regulation processes. Although initial empirical findings suggest that motivational regulation may also be relevant in situations of motivational conflict (e.g., Kim and Yu, 2024), little research has directly investigated whether motivational regulation strategies are associated with lower motivational interference in motivational conflict situations.

Existing motivational regulation strategies from the literature predominantly target the focal activity’s value or expectancy in order to help students increase and maintain their learning motivation (e.g., Engelschalk et al., 2015; Eckerlein et al., 2022). It may be assumed that established motivational regulation strategies are also relevant in situations of motivational conflict, as strengthening motivation for the focal task could help stabilize engagement despite competing alternatives. At the same time, motivational conflicts may require additional forms of motivational regulation, as they are characterized by competing alternative actions that remain motivationally relevant. The only exception among the established strategies that targets distracting alternative activities is environmental control, which aims to shield the learner from distracting alternatives. However, this strategy does not actively address the perceived attractiveness of those alternatives.

In 2007, Hofer (2007) raised the question of what strategies learners use to deal with motivational conflicts, a topic that remains under-researched. According to Hofer et al. (2017) there are three possible strategies that are particularly suitable for dealing with motivational conflicts: (1) *Linearization*, which involves arranging the competing activities in a sequence so that both can be realized. From the perspective of intergoal relations (Riediger and Freund, 2004), linearization might be interpreted as a way of converting conflicting goals into sequentially compatible ones. In this sense, it does not resolve the conflict by changing the relative importance of the goals or diminishing one of them, but by restructuring their temporal relationship. (2) *Compromise building* entails partially engaging in both activities to achieve a balance between them. Thus, it reflects an integrative form of regulation aimed at reducing conflict by partially satisfying competing goals. *Compromise building* might be helpful if goals facilitate each other, which would be consistent with research on intergoal facilitation and integration (Riediger and Freund, 2004). (3) *Reprioritization* might be regarded as a form of goal management in which the relative importance of competing goals is actively restructured (Cantor and Blanton, 1996; Kruglanski et al., 2002). Such (re-)prioritization may further support goal pursuit by inhibiting competing goals. A fourth possible conflict-related strategy, which complements the three strategies introduced above, is the downregulation of attractive action alternatives. Therefore, another strategy could be (4) the *devaluation of the alternative action*, which focuses on mentally reducing the attractiveness of the unchosen option. As high values of alternative actions are associated with higher levels of motivational interference (e.g., Grund and Fries, 2012), it should be fruitful to disparage the value of the unchosen alternative.

Established motivational regulation strategies primarily target the focal activity and rarely consider the motivational relevance of competing alternatives. It therefore remains unclear whether these strategies are sufficient in situations of motivational conflict. Although initial findings suggest that motivational regulation may be beneficial in such contexts (e.g., Kim and Yu, 2024), conflict-specific strategies that explicitly address alternative actions have not yet been systematically examined in academic settings. The present study therefore investigates whether, in addition to established strategies, conflict-related strategies such as *linearization*, *reprioritization, compromise building*, and *devaluation of the alternative* contribute to reduced motivational interference.

### 1.5 The present studies

Our studies examined the use of motivational regulation strategies (MRS) in the context of motivational conflicts and their association with motivational interference. Whereas Study 1 investigated established motivational regulation strategies, in Study 2 the strategies were complemented by additional strategies with focus on the motivational conflict. Thus, Study 2 functioned as replication and expansion of Study 1. According to the theory of motivational interference, stronger motivational conflicts impair motivation for the focal activity and increase interference in experience and behavior (Fries, 2006). Motivational regulation strategies (MRS) aim to enhance the motivational strength for learning tasks. A higher value for the focal task is associated to lower motivational interference (Brassler et al., 2016). A successful application of such strategies should therefore be associated with lower motivational interference. Prior research identifies strategy use, among other factors, as a positive predictor of regulation success (Engelschalk et al., 2017), suggesting that higher MRS use may help attenuate motivational interference.

**Hypothesis 1***:* In a motivational conflict, an extensive overall use of motivational regulation strategies is associated with a lower experience of motivational interference.

Engelschalk et al. (2017) also found that when the quality of the use of motivational regulation strategies is recorded in addition to the use of MRS, both the willingness to make an effort and regulatory success as well as the academic achievements of the learners could be predicted significantly better. With regard to motivational conflicts, we therefore also assume that a higher quality of use is associated with less motivational interference.

**Hypothesis 2**: In a motivational conflict, the higher the quality of the preferred motivational regulation strategy, the less motivational interference is experienced.

The motivation regulation strategies established so far (except the strategy of environmental control) pertain only to the focal action. We assume that there are other specific conflict-related strategies in addition to the established motivational regulation strategies that focus on non-chosen action alternatives and therefore on the presence of motivational conflicts. Are there additional strategies that have not been considered so far that work in the context of motivational conflicts? This question arises to examine potential differences in their deployment and the experience of motivational interference.

**Hypothesis 3a:** Both established action-related strategies and new conflict-related strategies are related with less motivational interference.

**Hypothesis 3b:** Conflict-related strategies significantly enhance the explanatory power regarding the variance in motivational interference above and beyond action-related strategies.

## 2 Study 1

### 2.1 Materials and methods of Study 1

In this scenario study, students were asked to imagine themselves in a given study-leisure conflict scenario and subsequently provided information on their use of motivational regulation strategies and their experience of motivational interference in that scenario.

#### 2.1.1 Participants

In this scenario study, 259 students participated, and after data cleansing, 214 of them were included in the analysis. Recruitment was carried out via a survey system of the university, survey networks and social networks. The participants’ age ranged between 18 and 48 (*M* = 23.54; SD = 4.26) and 78% identified themselves as female. A total of 44.9% of the participants studied psychology. The other participants studied in many different subject areas, including for instance: Teacher training (6.1%), Human medicine (4.2%), Mechanical engineering (3.7%), or Law (2.3%). A total of 87.4% of the participants had a grade point average of 2.5 or better. The average university semester was 6.44 (SD = 4.12) and 60.3% of the participants were aiming for a Bachelor’s degree at that time.

#### 2.1.2 Procedure

The data were collected using the *Qualtrics* survey tool. The participants first read a fictional scenario that represented a motivational conflict as a basis for answering all further questions. This is similar to the procedure used in previous research on motivational interference (e.g., Brassler et al., 2021, 2016, Dietz et al., 2005; Grund, 2013). At the beginning of the survey, all participants were informed about the objectives of the study, the voluntary nature of participation and the type of data processing and gave their consent. Requirements for participation were that participants were enrolled as university students and that they were fluent in German.

#### 2.1.3 Measures

The selected scenario in this study represented a motivational conflict between a learning activity (studying for an exam) and a leisure activity (watching a TV series), whereby both activities are typical actions in students’ everyday lives (Grund et al., 2014). The formulation was based on conflict scenarios from previous studies (Fries et al., 2008; Grund and Fries, 2012; Grund et al., 2014). The students were asked to imagine themselves in the described situation as accurately as possible. The English translation of the scenario is as follows: “Imagine you are sitting at your desk studying for an exam that will take place in a week. You have planned to study all afternoon today. At that moment, a friend writes to you to say that new episodes of your favorite series have come out today and they are supposed to be very exciting.”

The participants should then imagine that they had decided in favor of the learning activity and against the leisure activity.

The instruments which will be described in the following paragraphs consist largely of validated self-assessment questionnaires, whereby the wording of some items was adapted to the given scenario and individual newly constructed control items were integrated. Finally, socio-demographic information was requested.

##### 2.1.3.1 Scenario validation items

Three items were designed to assess the relevance to everyday life and the suitability of the selected scenario with regard to the research question. These items measured the extent to which participants were able to empathize with the situation, how likely it was to occur in their everyday study life and the extent to which it would elicit an experience of conflict. Another newly developed item assessed the extent of the motivation problem experienced in the scenario described (“*My motivation to continue learning is impaired in this situation*”). All items were rated on a six-point Likert scale (*1 = strongly disagree; 6 = strongly agree*).

##### 2.1.3.2 Use of motivational regulation strategies

In order to assess the use of specific MRS, the participants were asked how likely they would use certain strategies in the described scenario in order to motivate themselves to continue learning. Therefore, nine statements were presented in randomized order. The procedure of assessing each strategy with a single item was based on the study by Steuer et al. (2019). The selection of nine MRS was based on the validated questionnaire for recording motivational regulation (Engelschalk et al., 2015) (e.g., personal relevance: “*I try to make learning more relevant to me personally by making connections between the learning material and my own interests or experiences, e.g., by thinking about what I might need what I have learnt later in my appointment*”). For each of the nine items, the use of the described strategy was assessed on a six-point Likert scale (*1 = I would definitely not use this strategy; 6 = I would definitely use this strategy*). In addition, an open response field provided the opportunity to record further individual strategies.

##### 2.1.3.3 Application quality of motivational regulation strategies

Participants were asked to select their individually prioritized strategy in the scenario by ranking the available strategies. The quality of the selected strategy was assessed using a questionnaire consisting of five items with a six-point Likert scale (*1 = strongly disagree; 6 = strongly agree*) (Engelschalk et al., 2017). For example, one item on the scale was: “*I adapt this strategy if it is not working properly*.” With α = 0.81, the scale showed good internal consistency.

##### 2.1.3.4 Experience of motivational interference

Motivational interference was assessed using a scale based on Grund et al. (2014). The instrument captures impairments in experience and behavior during the focal learning activity, including affective, cognitive, and behavioral impairments. The scale comprised 20 items, with four items each measuring the facets of *mood*, *distractibility*, *thoughts about the alternative*, *persistence/switching*, and *depth of processing*. For example, an item in the depth of processing facet is: “*I am not particularly thorough when learning*.” Answers were given on an eight-point Likert scale (1 = *not at all true; 8 = exactly true*). The internal consistency was α = 0.93. Higher values indicated stronger motivational interference.

### 2.2 Data analysis

Data were analyzed *using IBM SPSS Statistics*. An overall index of the nine MRS was formed for the use of MRS. Linear and multiple regressions were calculated to test the hypotheses. The use of MRS and quality of MRS use were defined as independent variables and motivational interference as the dependent variable. Correlations between individual MRS and motivational interference were also calculated.

### 2.3 Results of Study 1

Descriptive statistics and bivariate correlations among variables are displayed in Table 1. As shown in Table 1, greater overall use of motivational regulation strategies was associated with lower motivational interference. The subjective experience of a motivational conflict was *M* = 3.77, *SD* = 1.49.

**TABLE 1:** Descriptive statistics and bivariate correlations among key variables.

Variables	*M*	*SD*	2	3
1. Use of MRS	4.21	0.63	0.44**	−0.33**
2. Application quality of MRS use	3.80	1.02	–	−0.29**
3. Motivational interference	3.96	1.11	–	–

*N* = 214. MRS, motivation regulation strategies.

**P < 0.01.

The results show that the mean level of the use of MRS is clearly higher than the theoretical midpoint of 3.5. This suggests that students actually use motivational regulation strategies in the context of a motivational conflict.

A higher use of MRS is associated with lower motivational interference, so that Hypothesis 1 can be confirmed. It was found that the use of MRS as a predictor explains 11% of the variance of the motivational interference [*R*^2^ = 0.11 adjusted *R*^2^ = 0.10 *F*(1, 212) = 27.91, *p* < 0.001]. According to Cohen (1988), this corresponds to a moderate explanation of variance. As expected, the association was negative (β = −0.33), whereby the strength of the association can be interpreted as medium (Schäfer, 2016).

As shown in Table 2, most motivational regulation strategies are negatively correlated with motivational interference. In particular, *proximal goal setting* and *environmental control* appear to be associated with lower levels of motivational interference.

**TABLE 2:** Bivariate correlations among specific strategies use and motivational interference.

Specific strategies	*M*	*SD*	Motivational interference
1. Enhancing personal significance	3.74	1.32	−0.18**
2. Enhancing of situational interest	3.26	1.35	−0.10
3. Mastery self-talk	3.63	1.40	−0.19**
4. Performance-approach self-talk	4.43	1.32	−0.24***
5. Performance-avoidance self-talk	4.05	1.48	0.10
6. Ability-focused self-talk	4.05	1.32	−0.18**
7. Self-consequating	4.89	1.14	−0.19**
8. Proximal goal setting	5.07	.91	−0.27***
9. Environmental control	4.78	1.31	−0.30***

*N* = 214.

**p < 0.01,

***p < 0.001.

In Hypothesis 2, the assumption was made that the higher the quality of the prioritized strategy used, the less interference is experienced in a motivational conflict. The relation was negative as expected. It was shown that quality as a predictor could explain 8% of the variance of the motivational interference criterion [*R*^2^ = 0.08; *F*(1, 201) = 17.98, *p* < 0.001].

### 2.4 Brief discussion of Study 1

Results of Study 1 suggest that students report that they would use motivational regulation strategies in a scenario of motivational conflict. The use and also the application quality of MRS were negatively associated with the experience of motivational interference. Specifically, students who reported greater use and higher-quality application of strategies experienced lower levels of interference in the conflict situation.

On the level of single MRS there were two strategies that were non-significant: performance-avoidance self-talk and enhancing of situational interest. Actually, although performance-avoidance self-talk has been in the set of motivational regulation strategies for a long time (Schwinger et al., 2009), it has been discussed in recent research that it might no longer be considered part of the set of adaptive MRS (Schwinger and Otterpohl, 2017).

*Enhancing of situational interest* in contrast had been functional MRS in other contexts. This finding supports the assumption that the set of established MRS might not be sufficient to regulate motivation in all kinds of situations (see also, Eckerlein et al., 2022).

Established strategies mainly focus on strengthening motivation for the focal action. It is likely that motivational conflicts, which are characterized by the simultaneous activation of competing actions, may pose specific regulatory demands to address the conflict between concurring actions (see also, Trautner et al., 2025). Study 2 therefore extends the approach of Study 1 by suggesting additional motivational regulation strategies that are specifically related to the regulation of motivational conflicts.

## 3 Study 2

### 3.1 Design and procedure of Study 2

Study 2 builds on Study 1 in methodology and content, thus, while describing Study 2 redundant information will be omitted and we focus on describing changes and additions.

#### 3.1.1 Participants

In Study 2, 242 students took part in the survey. The students’ age ranged between 18 and 49 (*M* = 23.00, *SD* = 4.43). A total of 84.3% of the participants were female, 71.5% studied psychology. The other participants studied in many different subject areas, e.g., Teacher training (11.6%), Business Psychology (1.7%). A total of 84.6% had a current grade point average of 2.5 or better. The average number of semesters spent at university was 5.49 (*SD* = 3.34) and 83.9% of participants were currently aiming for a bachelor’s degree.

#### 3.1.2 Procedure

As in Study 1, data for the scenario study were collected using the *Qualtrics* survey tool. The participants were presented with the same scenario, which dealt with a potential motivational conflict between studying for an exam and watching their favorite series. The framework and conditions of the survey are comparable to Study 1. Study 2 was also approved by the ethics committee of Bielefeld University (2021-285) prior to the start date of the survey.

#### 3.1.3 Measures

For Study 2, measurement instruments used in Study 1 were adopted and supplemented with several additional and newly developed items for the motivational regulation strategies scale. Specifically, we included four new conflict-related strategies that directly address the conflict and focus on both conflicting actions.

As proposed by Hofer et al. (2017), we included additional strategies for dealing with motivational conflicts. The item for *linearization* was “I organize the activities in a sequence so that I can do both (e.g., by studying first and then watching the series).” The item for *reprioritization* was “I make it clear to myself which activity is more important to me at the moment and thus set a priority (e.g., by telling myself that learning has a higher priority than watching the series at the moment).” The item for *compromise building* was “I make a compromise (e.g., by only learning a little bit and only watching half an episode or one episode of my series) so that I can have a bit of both activities.” Finally, we added one additional strategy for the downregulation of attractive alternatives (see also, Trautner et al., 2025) the item for *devaluation of the alternative action* was “I think about reasons not to watch the series (e.g., by telling myself that the series won’t help me achieve my goals).”

All the other measures used are identical to Study 1. The internal consistency of the motivational interference scale was α = 0.92.

#### 3.1.4 Data analysis

The data was processed and analyzed using *IBM SPSS Statistics*. An overall index of the nine established MRS was formed to replicate the findings of Study 1. To test the hypotheses, correlations were calculated in order to determine relationships between the use of motivational regulation strategies (independent variables) and motivational interference (dependent variable). In addition, a hierarchical regression analysis was conducted to test whether the newly defined conflict-related strategies *linearization*, *reprioritization*, *compromise building*, and *devaluing the alternative action* explain additional variance in motivational interference beyond the known strategies and therefore should be considered in future research.

### 3.2 Results of Study 2

In Table 3 descriptive statistics and bivariate correlations among the key variables are presented. As expected, the use of MRS and the application quality of MRS use were both negatively related to motivational interference. The subjective experience of a motivational conflict was *M* = 4.91, *SD* = 1.20.

**TABLE 3:** Descriptive statistics and bivariate correlations among key variables.

Variables	*M*	*SD*	2	3
1. Use of MRS	4.20	0.62	0.35**	−0.29**
2. Application quality of MRS use	3.95	1.00	–	−0.34**
3. Motivational interference	4.03	1.07	–	–

*N* = 242. MRS, motivation regulation strategies.

**P < 0.01.

The descriptive statistics of the specific MRS and the bivariate correlations among the strategies and the experience of motivational interference are displayed in Table 4.

**TABLE 4:** Bivariate correlations among specific strategies and motivational interference.

Specific strategies	*M*	*SD*	Motivational interference
1. Enhancing personal relevance	3.76	1.35	−0.06
2. Enhancing of situational interest	3.24	1.47	−0.11
3. Mastery self-talk	3.43	1.35	−0.26**
4 Performance-approach self-talk	4.58	1.36	−0.15*
5. Ability-focused self-talk	3.89	1.40	−0.28**
6. Performance-avoidance self-talk	4.48	1.33	−0.03
7. Environmental control	4.57	1.30	−0.24**
8. Self-consequating	4.95	1.29	−0.26**
9. Proximal goal setting	4.73	1.18	−0.26**
10. Linearization	4.88	1.16	−0.20**
11. Compromise building	3.82	1.54	0.35**
12. Reprioritization	4.81	1.19	−0.36**
13. Devaluation of the alternative action	3.48	1.51	−0.06

*N* = 242.

*P < 0.05,

**p < 0.01.

A higher use of MRS was associated with lower motivational interference, so that hypothesis 1 can be confirmed.

The use of MRS as a predictor explained 35% of the variance of the motivational interference [*R*^2^ = 0.35, adjusted *R*^2^ = 0.31; *F*(13, 228) = 9.38, *p* < 0.001].

Table 5 reports the results of the hierarchical regression analysis predicting motivational interference. The standardized regression coefficients for the established strategies (Model 1) and the additionally included conflict-related strategies (Model 2) are presented, showing that several established strategies are negatively associated with motivational interference, while in Model 2 *compromise building* (positive) and *reprioritization* (negative) emerge as additional predictors.

**TABLE 5:** Hierarchical regression of established strategies and additional conflict-related strategies on motivational interference.

Standardized regression coefficients	Model 1^1^	Model 2^2^		
Model 1^1^	ß	ß	T	Sig.
Constant	6.38	–	14.83	<0.001
1. Enhancing personal relevance	0.10	–	1.48	0.140
2. Enhancing of situational interest	0.02	–	0.32	0.751
3. Mastery self-talk	−0.15	–	−2.13	0.034
4 Performance-approach self-talk	−0.03	–	−0.41	0.679
5. Ability-focused self-talk	−0.12	–	−1.81	0.072
6. Performance-avoidance self-talk	0.02	–	0.34	0.732
7. Environmental control	−0.13	–	−1.97	0.050
8. Self-consequating	−0.16	–	−2.51	0.013
9. Proximal goal setting	−0.17	–	−2.77	0.006
**Model 2^2^**				
Constant	–	5.98	13.56	<0.001
1. Enhancing personal relevance	–	0.11	1.88	0.061
2. Enhancing of situational interest	–	−0.08	−1.36	0.174
3. Mastery self-talk	–	−0.08	−1.27	0.205
4 Performance-approach self-talk	–	0.02	0.29	0.771
5. Ability-focused self-talk	–	−0.13	−2.04	0.043
6. Performance-avoidance self-talk	–	0.01	0.18	0.856
7. Environmental control	–	−0.10	−0.17	0.096
8. Self-consequating	–	−0.08	−1.27	0.205
9. Proximal goal setting	–	−0.12	−2.04	0.043
10. Linearization	–	−0.07	−1.05	0.294
11. Compromise building	–	0.34	6.07	<0.001
12. Reprioritization	–	−0.25	−3.90	<0.001
13. Devaluation of the alternative action	–	0.03	0.58	0.563

Dependent variable: motivational interference. MRS, motivational regulation strategies.

^1^Predictor variable Model 1: established strategies.

^2^Predictor variable Model 2: established and new strategies.

### 3.3 Brief discussion of Study 2

In Study 2 we could replicate the negative association between the use of MRS and the application quality of MRS and motivational interference. On the level of the specific MRS, again, *enhancing of situational interest* was not significantly related to motivational interference. Also, the strategy *enhancing personal relevance* was not significantly related to motivational interference, which is, like *enhancing of situational interest*, another value-related strategy. This finding raises the question of whether value-based strategies may not optimally suit situations of motivational conflict, in which an attractive alternative action competes with the focal task. The new conflict-related strategies turned out to be quite heterogeneous in the relation with motivational interference. *Reprioritization* and *linearization* were related to less motivational interference and therefore beneficial strategies. The effect of *reprioritization* remained stable even when controlling for all established and new strategies and can therefore be interpreted as a specifically useful strategy. In contrast, devaluation of the alternative action turned out to be not significant. *Compromise building* was even positively correlated with motivational interference, which indicates that this strategy might rather increase than decrease motivational interference and therefore may be seen as a less adaptive strategy in dealing with motivational conflicts.

The missing effect of *linearization* (after controlling for the other MRS) might have occurred due to offering the strategy *reprioritization* – which makes *linearization* unnecessary. Hence, further research using other conflict scenarios and methodological approaches in which competing strategies are not presented within the same scenario may shed light on the functionality of MRS in motivational conflict situations.

## 4 General discussion

The present studies examined the role of motivational regulation strategies in situations of motivational conflict, a distinct type of motivational problem arising from competing alternative actions. Such conflicts are particularly relevant because they are often associated with motivational interference, that is, impairments in experience and behavior during the focal activity (e.g., Hofer and Fries, 2016). The present studies investigated how established motivational regulation strategies and additionally introduced conflict-related strategies are associated with motivational interference in such situations.

Across two scenario studies, both the overall use of motivational regulation strategies and the quality of strategy application were negatively related to motivational interference. This is in line with previous findings by Kim and Yu (2024) which implied that motivational regulation strategies can support school students within motivational conflicts. With our work we were not only able to replicate the negative association between motivational regulation strategies and motivational interference. We also could extend previous findings to the population of university students.

Furthermore, by examining individual MRS rather than only a global indicator of motivational regulation, our studies allow a much more differentiated view on how specific strategies are related to motivational interference. Specifically, most established strategies (e.g., *mastery self-talk*, *ability-focused self-talk*, *environmental control*, *self-consequating*, *proximal goal setting*) were negatively related to motivational interference. In contrast, *Performance-avoidance self-talk*, *value-enhancing, enhancing situational interest* and *enhancing personal relevance* strategies showed no significant associations. This pattern suggests that although many established strategies remain beneficial in motivational conflicts, not all forms of regulation are equally effective in this context.

In Study 2 we extended the set of motivational regulation strategies by adding four conflict-related strategies. Three of these strategies - *linearization*, *reprioritization*, and *compromise building -* were derived from theoretical considerations on motivational action conflicts (Hofer et al., 2017). In addition, we introduced *devaluation of the alternative action* as a further strategy that directly targets the attractiveness of the competing alternative. This is in line with the idea that in motivational conflicts, strategies of downregulation of alternatives may play a role (Trautner et al., 2025).

Results showed a heterogeneous pattern: *Reprioritization* and *linearization* actually were associated with lower levels of motivational interference. A reason for this may be the own conscious decision for a specific course of action. This could be interpreted as enacted autonomy in self-determination theory (Deci and Ryan, 2000) which contributes to increased motivation. These results point to the fact that it is worth considering conflict-related strategies, especially reprioritization, as motivation regulation strategies as they have explanatory value above and beyond the classical MRS.

*Devaluation of the alternative action* was not significantly related to motivational interference at all although previous research showed that the value of an action alternative is clearly associated with higher impairments in a focal task (e.g., Brassler et al., 2016; Grund et al., 2014). One possible explanation is that attempting to devalue an alternative does not necessarily lead to an actual reduction in its perceived value. Instead, directing attention toward the alternative might even increase its salience for some students. From the perspective of the approach–avoidance framework (Elliot, 2006), such strategies may resemble avoidance-focused regulation attempts that focus individuals toward avoiding the undesired option rather than directing attention and resources toward the focal task.

The strategy *compromise building* was even positively related to motivational interference. One explanation for this finding could be that *compromise building* resembles a form of multitasking. Prior research has shown that multitasking is often associated with reduced performance and motivational problems (e.g., Kirschner and De Bruyckere, 2017). In such situations, individuals attempt to engage in two competing activities simultaneously, which may prevent full engagement in either activity. As a consequence, both actions remain cognitively activated and compete for limited attentional resources, which may increase the likelihood of experiencing motivational interference (e.g., Masicampo and Baumeister, 2011). Besides cognitive impairments, compromise building may entail emotional costs, as individuals may perceive both goals as only partially fulfilled. Decision-making research suggests that trade-offs and compromise solutions are often experienced as less satisfying than clear goal prioritization (Simonson, 1989; Shafir, 1993). Taken together, in contexts characterized by largely incompatible goals, *compromise building* may reduce experienced conflict but at the expense of weakened commitment to both goals, ultimately resulting in suboptimal outcomes. Thus, based on the present findings, *compromise building* does not appear to represent an adaptive strategy for dealing with motivational conflicts.

Interestingly, in general, strategies that focused on value parameters, were not associated with lower motivational interference in our scenario. One possible explanation is that in our motivational conflict scenario, the focal task may already be perceived as relatively important. As a consequence, strategies that aim to further increase task value may have limited potential to make a difference for motivational dynamics of the situation. Furthermore, motivational conflicts are characterized by the presence of an attractive alternative. Thus, the value of the competing action, rather than the value of the focal task itself, may be more salient and therefore central for the emergence of motivational interference in such situations. In contrast, strategies targeting success expectations for the focal task may help stabilize engagement in the chosen activity. When students feel more confident about successfully completing the focal task, competing alternatives may be experienced as less disruptive.

In sum, the present findings provide three main insights. First, established motivational regulation strategies appear to be beneficial not only for motivational problems related to the focal task but also in situations of motivational conflict. Second, the associations of these strategies are not uniform, as some strategies showed no relation to motivational interference in the present studies. Third, the results suggest that strategies that directly address the motivational conflict itself may play an additional role in reducing motivational interference.

Therefore, our research contributes to both the fields of motivational conflict and interference and motivational regulation. First, motivational interference appears to be a problem that can be actively addressed through self-regulation. It would be worthwhile to focus on options that help reducing negative effects of motivational conflicts in students’ lives.

Furthermore, our findings contribute to a more differentiated understanding of motivational regulation problems. Previous research has primarily distinguished between expectancy-related and value-related motivational problems (e.g., Engelschalk et al., 2015; Eckerlein et al., 2022), both of which refer to characteristics of the focal learning activity. In contrast, the present studies focused on motivational conflicts that arise when an attractive alternative action competes with the focal task. In these situations, motivation is not necessarily impaired because the focal task is perceived as unimportant or too difficult, but because an alternative action remains motivationally active. From this perspective, motivational conflicts may represent a third important class of motivational regulation problems. Future research could investigate this idea more systematically by comparing different types of motivational problems (e.g., value-related, expectancy-related, and conflict-related) and investigating which strategies are most effective for each type of motivational problem.

Additionally, results indicate that the various motivational regulation strategies do not represent a homogeneous scale, as already reported in previous studies (e.g., Schwinger et al., 2007, 2009), particularly with regard to the newly considered conflict-related strategies. This indicates that different strategies may have different modes of action and conditions of application. The results suggest that conflict-related strategies may have specific contextual factors or individual differences in application and effectiveness. It could therefore be useful to analyze specific strategies in more detail in order to gain a deeper understanding of how they are related to student motivation. Such an approach could help to better understand the dynamics of motivational regulation and to develop specific interventions that are tailored to the needs of learners.

When considering the individual motivational regulation strategies, several implications can be made. One question that arises is the question how to categorize motivational regulation strategies. In previous literature, there has been the attempt to differentiate between strategies that are beneficial for expectancy problems from those that are beneficial in facing value problems (e.g., Eckerlein et al., 2022). In both cases, the focus was on strengthening the focal task. Our research shows that it would be worthwhile to also consider concurring action alternatives. Therefore, we suggest to also consider conflict-related strategies. Actually, one of the establishes motivation regulation strategies already fits to this category, that is, environmental control. Our research shows that also *reprioritization* and *linearization* might help. Research on motivational interference even provided first indications that expectancies regarding concurring activities may be related to motivational interference in a focal task (Brassler et al., 2021). This may point to the relevance of further investigating possible motivational regulation strategies targeting expectancies of competing alternatives. Furthermore, *performance-avoidance self-talk* was not related to lower motivational interference. As mentioned before, this strategy has been recently discussed as generally maladaptive, since research has shown that it is ineffective in regulating students’ learning motivation (see Schwinger and Otterpohl, 2017; Eckerlein et al., 2019).

### 4.1 Limitations and future research

Besides the contributions of the present studies, several limitations should be acknowledged. These limitations point to promising directions for future research.

The use of a standardized scenario offered the advantage of a high degree of comparability within the sample, as all students responded to the same motivational conflict situation, compared to other approaches where individual conflicts are assessed (e.g., with experience sampling, e.g., Grund et al., 2015a; Hofmann et al., 2012).

However, the use of a single fictional scenario entails several limitations. First, the generalizability of the findings may be restricted, as it remains unclear to what extent the selected scenario adequately represents motivational conflicts that students encounter in everyday life. Although the scenario validation items indicated that, on average, participants agreed that the situation reflected a motivational conflict (see Tables 1, 3), this does not guarantee that the emotional intensity, motivational dynamics, and situational complexity of real-life conflicts were fully captured. Future research should nonetheless investigate our research questions with different scenarios to find out if our findings can be generalized to other motivational conflicts in students’ lives. Furthermore, this can shed light on which strategies are most beneficial for which kinds of conflicts. Second, responses to hypothetical scenarios may not fully correspond to actual behavior in real conflict situations. Scenario-based responses are more likely to reflect intentions, evaluations, or self-attributions rather than concrete behavior under real conditions. In addition, responses may be influenced by participants’ psychological lay theories about how they believe they would think, feel, or act in such situations (Brassler et al., 2016, 2021; Fries et al., 2008). A further potential source of bias is social desirability, as participants may have preferred socially acceptable or morally appropriate responses. Although prior research suggests that scenario methods can serve as a reasonable proxy for actual experience and behavior, these concerns highlight the need for methodological triangulation. Previous research on motivational conflicts used different approaches which showed that we can assume that the scenario method is a good proxy for the actual experience and behavior, but it will be important to investigate this in future research. Future studies should therefore complement scenario-based approaches with more behaviorally oriented methods, such as experience sampling methodology (e.g., Csikszentmihalyi and Larson, 1987; Grund et al., 2015a; Zirkel et al., 2015), to assess motivational conflicts and corresponding experiences directly in real time and natural contexts.

In our concrete conflict scenario, it was not clearly defined what would be the consequences if one would postpone the learning activity. Therefore, we do not know if *linearization* would be an adequate means to achieve both the learning and the leisure activity or if postponing the learning activity would automatically lead to less time for learning. Future research should systematically investigate if there are differences if the concurring activity can be postponed or not. According to Hofer (2004), motivational conflicts arise when several activities that cannot be carried out simultaneously are rated highly at a certain point in time, e.g., due to a lack of time. It is not clearly defined whether the actions can be performed one after the other, as in our studies, so that the *linearization* strategy can be used, or whether one must finally decide on an action in a motivational conflict.

A further limitation of our study is the use of a single item to assess each individual MRS. Single-item measures can offer a time-efficient and resource-conscious approach, reducing the time and effort required from participants and potentially decreasing repetitiveness, which may enhance participation rates and response quality (see Allen et al., 2022). This is particularly relevant given the large number of MRS assessed in the present study. However, single-item measures are associated with methodological limitations: reliability may be constrained because internal consistency cannot be estimated and measurement error cannot be attenuated through the aggregation of multiple items. This may also compromise construct validity, as more complex or multidimensional aspects of a construct cannot be captured in a differentiated manner (see Diamantopoulos et al., 2012). Accordingly, the findings should be interpreted with caution in light of these measurement-related limitations.

In our study, participants were asked to select a single preferred strategy. In future research, this operationalization could be extended to capture both the simultaneous use of multiple strategies and the sequential application of different strategies over time. Such an approach would allow for a more comprehensive understanding of motivational regulation processes, as students in real motivational conflicts may employ multiple strategies in parallel or dynamically switch between strategies depending on situational demands. Longitudinal designs would be particularly valuable here in order to analyze developmental processes and to record the temporal dynamics of motivational regulation processes in a more systematic way. Experimental designs would also be conceivable in order to investigate the causal effects of specific strategies more in detail. Although further refinement is warranted, the present study offers a meaningful and empirically grounded starting point for future research on motivational regulation processes.

Our paper is primarily grounded in the expectancy-value theory, in which motivational interference can be conceptualized as a cost component. Complementing this perspective, future research could systematize motivational regulation strategies in conflict situations using theory-guided taxonomies, for example from a self-determination theory (SDT) perspective. Ahmadi et al. (2023), for instance, developed a comprehensive and systematically derived classification of teacher behaviors based on their function in supporting basic psychological needs. A similar SDT-informed approach could be applied to motivational regulation strategies. For example, mastery self-talk could be conceptualized as promoting the experience of competence and thus be assigned to the basic need for competence.

### 4.2 Practical implications

The present findings also have several practical implications for supporting students in dealing with motivational problems during learning.

Two application contexts appear particularly relevant. First, the results provide insights into how students can be supported when motivational conflicts arise during studying. Whereas prior research has primarily described the phenomenon and its consequences, less attention has been paid to how students can effectively cope with such situations. The present findings contribute to this question by linking the experience of motivational conflicts to the use of motivational regulation strategies. Because motivational regulation strategies have previously been shown to support students in dealing with motivational problems during learning, they represent a promising approach for coping with motivational conflicts. Although future research is needed to support our results, our findings indicate, that certain types of strategies may be particularly relevant in such situations. In particular, expectancy-related strategies appear to be associated with more favorable experiences of motivational interference. Strengthening students’ expectations of success may therefore help them maintain engagement with the focal learning activity despite the presence of attractive alternatives. In addition, strategies that explicitly address the conflict between competing activities may also be beneficial. Such strategies include, for example, actively reflecting on reasons for prioritizing the learning task or structuring the environment in ways that reduce competing temptations (e.g., environmental control). Overall, supporting students in recognizing motivational conflicts and applying appropriate strategies may help protect their learning in situations of competing goals.

Beyond motivational regulation strategies, other approaches may also support students in dealing with motivational conflicts. For example, mindfulness-based approaches may help students become more aware of emerging motivational conflicts and their associated impulses. Increased awareness may allow students to notice these conflicts earlier and respond more deliberately rather than automatically shifting their attention to alternative activities. Although empirical research in this area is still limited, initial findings suggest a relationship between mindfulness and the experience of motivational interference (e.g., Grund et al., 2021), indicating that this may be a promising direction for future practical interventions.

However, the present considerations primarily address how students can deal with motivational conflicts once they have already emerged. An additional perspective concerns the prevention of such conflicts in the first place. From this viewpoint, it may be beneficial to support students in structuring their study routines in ways that reduce the likelihood of competing goals arising during learning. Previous research has already provided initial findings in the area of habit building and the reduction of motivational interference (Stojanovic et al., 2020).

Also here, mindfulness may also play a preventive role. By fostering greater awareness of one’s goals, priorities, and everyday routines, mindfulness practices may help students organize their study environments and schedules in ways that minimize potential conflicts between studying and alternative activities. Supporting students not only in coping with motivational conflicts but also in preventing them may therefore represent a promising direction for practical interventions aimed at improving students’ learning experiences (Grund and Senker, 2018).

The metamodel of motivational regulation according to Miele and Scholer (2018) and a study by Steuer et al. (2019) support the assumption that knowledge about motivational regulation is necessary in order to be able to use motivational regulation strategies effectively.

In two subsequent training studies Steuer et al. (2026) were able to demonstrate that training in motivational regulation strategies (MRS) for university students is effective. It appears to be crucial to provide both declarative strategy knowledge and knowledge about possible ways to enhance the quality of strategy application. In addition, a third aspect is essential: the conditional knowledge of the fit between the specific MRS and the actual motivational problem. Our studies suggest that motivational conflicts, as another type of motivational problem, in addition to expectancy and value, need to be addressed by different MRS. Our studies provide the empirical base to broaden the scope of a training as described above and thus help students successfully cope with a broader range of motivational problems.

### 4.3 Conclusion

Motivational conflicts are a common feature of students’ learning situations, as studying often takes place in contexts where multiple attractive activities compete for time and attention. The present research suggests that motivational regulation strategies are meaningfully related to how students deal with such conflicts, as their use and quality were associated with lower motivational interference during learning. By extending motivational regulation research to situations in which motivational difficulties arise from competing alternatives rather than from the focal task itself, the findings broaden current perspectives on how students regulate their motivation in everyday learning contexts.

## Data Availability

The raw data supporting the conclusions of this article will be made available by the authors, without undue reservation.
